# Patient-centered prescription opioid tapering in community outpatients with chronic pain: 2- to 3-year follow-up in a subset of patients

**DOI:** 10.1097/PR9.0000000000000851

**Published:** 2020-09-17

**Authors:** Maisa Ziadni, Abby L. Chen, Parthasarathy Krishnamurthy, Pamela Flood, Richard L. Stieg, Beth D. Darnall

**Affiliations:** a Division of Pain Medicine, Department of Anesthesiology, Perioperative and Pain Medicine, Stanford University School of Medicine, Palo Alto, CA, USA; b Department of Marketing and Entrepreneurship, CT Bauer College of Business, University of Houston, Houston, TX, USA; c LLC, Frisco, CO, USA

**Keywords:** Opioid tapering, Chronic pain, Pain intensity, Opioid use, Patient-centered

## Abstract

**Objective:**

We previously reported promising results for a 4-month patient-centered voluntary opioid tapering study. Key questions remain about the durability of effects and possible risks after opioid reduction. We provide the longest follow-up data to date for prospective opioid tapering: 2- to 3-year follow-up for pain intensity and daily opioid use in a subset of patients from our original 4-month opioid tapering study.

**Methods:**

Twenty-three patients (44% of original sample) responded to contact attempts through telephone and reported their average pain intensity and current opioid use. Opioid doses were converted to morphine equivalent daily dose (MEDD). Data were analyzed within a repeated-measures model where time (baseline, 4 months, and 2–3 years) was the within-subject factor.

**Results:**

Among reachable patients, the effect of time on change in MEDD from baseline to 4 months to 2 to 3 years was significant. Since baseline, 20 (95%) of the current sample reduced MEDD, and 15 (71%) further reduced MEDD at 2- to 3-year follow-up. There was no effect of time on change in pain intensity from baseline to 4 months to 2 to 3 years. Since baseline, 11 (52%) of the current sample reported pain reduction, and 12 (57%) reported reduced pain from the 4-month follow-up to the 2- to 3-year follow-up. Five (24%) reported increased pain intensity.

**Conclusion:**

Study findings reveal continued MEDD reduction and enduring pain stability 2 to 3 years after a patient-centered voluntary opioid tapering program for a substantial fraction of patients. Notably, we were not able to verify current opioid use through medical records and were limited by self-report.

## Abstract 2

Study findings reveal continued opioid reduction and enduring pain stability for a substantial fraction of patients, 2 to 3 years after a patient-centered voluntary opioid tapering program.

## 1. Introduction

Research on prescription opioid tapering is scant, low in quality, and minimal data exist for tapering outcomes exceeding 1 year.^2,8,12^ Most data exist for intensive, inpatient and outpatient programs, with follow-up timepoints that do not exceed 1 year.^2,12^ For example, a study by Huffman et al.^8^ reported retrospective analysis of data for patients with chronic pain after an intensive outpatient interdisciplinary pain management program, showing improvements in opioid cessation, but return-to-use rates were 10.5% at 6 months and 30.7% at 1 year. Recent work also indicates increased risks of opioid overdose and suicide for veterans after opioid tapering.^10^ More recently, a study by Nicholas et al.^9^ showed that after cognitive-behavioral therapy-based interdisciplinary treatment for chronic pain, the use of opioids was significantly reduced, and these gains were maintained over the 12-month follow-up.

Better data are needed to understand how tapering methodology—including voluntary vs involuntary patient participation—impacts safety and outcomes. We previously reported results for a 4-month voluntary patient-centered opioid tapering study in 51 patients from Colorado.^3^ Patients with noncancer chronic pain taking long-term opioids at community suburban and rural pain clinics were provided education about the benefits of opioid reduction (reduced health risks without increased pain) by their prescribing physician. Physicians offered to partner with patients to slowly reduce their opioids and arrive at their lowest comfortable dose at 4 months. Patients were able to control the pace of their taper, pause their taper, and stop their taper if they wished; importantly, the taper progam was not unidirectional, thereby allowing physicians to address the needs of the individual patient. Patients reported average morphine equivalent daily dose (MEDD) decreases of roughly 50% at 4 months with stable pain intensity. Although promising, questions remain about the durability of these effects. Furthermore, detailed data are lacking for individual long-term taper outcomes. The current study aimed to report long-term outcomes on pain intensity and daily opioid use for the subset of voluntarily tapered patients we were able to contact.

## 2. Methods

Using email and telephone outreach up to 3 years later, we made contact with 44% of our original sample (M = 156 weeks, SD = 36.72; range = 98–203 weeks) and 1 additional patient who was not reached at the initial follow-up. Brief telephone interviews were used to collect average pain intensity and current opioid use. Average pain intensity was assessed by asking patients, “In the past 7 days, how intense was your average pain?” on a scale from 0 (no pain) to 10 (the worst pain imaginable). Current opioid use was assessed by initially asking patients a binary (yes/no) question, “Are you currently taking opioid medication?” followed by a drop-down menu of opioid medication options, including the respective dose options, frequency, and total milligrams taken per day.

All patients had new prescribers at the time of this survey, and medical records were unavailable. There were no Colorado death records for the unreachable 29 patients. Study procedures were approved by the Stanford University Institutional Review Board.

We converted opioid doses to MEDD using the conversion guidelines provided by the Centers for Medicare and Medicaid Services. Change in MEDD from baseline was the primary outcome, and pain intensity was secondary. Data were analyzed within a repeated-measures model where time (baseline, 4 months, and 2–3 years) was the within-subject factor. We hypothesized continued reduction in MEDD over time with stable pain report.

## 3. Results

Among reachable patients (n = 23), average age (SD) was 51 (13) years, and 11 (48%) were women. Twenty-nine of 52 enrolled patients (56%) did not respond to contact attempts and therefore are not included in this follow-up report. However, 2 of the patients were on suboxone and were therefore excluded from follow-up analyses involving change in MEDD and pain intensity. Table 1 provides characteristics and results. No variables predicted study completion.

**Table 1:** Characteristics and outcomes.

Variable	Reachable patients* (n = 23)				Unreachable patients† (n = 29)	
	Baseline	4 mo	2–3 y		4 mo	
	Mean (SD)	Mean (SD)	Mean (SD)	*P*	Mean (SD)	*P*
Opioid dose*‡	298.8 (241.0)	147.0 (124.0)	65.2 (92.1)	0.0001§	246.4 (36.30)	0.07‖
Pain intensity*	4.6 (2.2)	4.1 (2.0)	3.9 (2.1)	0.48§	5.17 (0.40)	0.13‖
Pain catastrophizing	20.4 (11.3)	14.1 (10.8)				
Fatigue**	59.4 (10.4)	57.9 (11.9)				
Anxiety**	58.8 (10.5)	54.8 (8.2)				
Depression**	56.8 (11.0)	53.6 (8.6)				
Sleep disturbance**	62.5 (9.7)	59.0 (10.8)				
Pain interference**	63.8 (8.4)	61.4 (8.7)				
Pain behavior**	60.5 (4.9)	59.9 (5.3)				
Physical function#**	37.6 (5.4)	38.6 (7.2)				

* Reachable patients provided 2- to 3-year follow-up data. Two patients were removed from primary analyses involving opioid dose and pain intensity due to taking suboxone.

† Did not respond to contact attempts and did not provide 2- to 3-y follow-up data.

‡ Opioid dose (morphine equivalent daily dose).

§ The *P*-value represents the main effect of time (the repeated-measures factor).

‖ Least-significant means report with standard error parentheses; *P*-value represents the comparison between reachable and unreachable patients at 4 mo on the primary variables of interest.

# Lower scores reflect worse function.

** Patient-Reported Outcomes Information System (PROMIS) measure.

**Figure 1.**
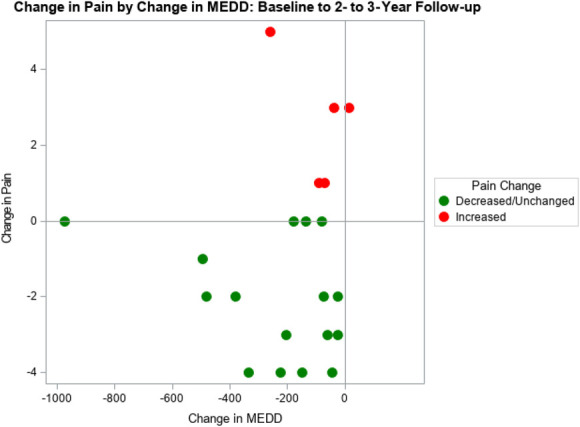
Change in opioid morphine equivalent daily dose and change in pain intensity score from baseline to 2- to 3-year follow-up for reachable patients (n = 21)*. *Two patients were removed from primary analyses due to taking suboxone.

The effect of time on change in MEDD from baseline to 4 months to 2 to 3 years was significant (*P* < 0.0001); the reduction in MEDD from 4 months (mean = 147.04, SE = 25.86) to 2 to 3 years (mean = 66.59, SE = 19.94) was significant (*P* = 0.012). Similarly, the reduction in MEDD from baseline to 2- to 3-year follow-up was significant (*P* < 0.0001) (Fig. 1). Since baseline, 20 patients of the current sample of 21 (95%) reduced MEDD by the 2- to 3-year follow-up, and 15 patients of the current sample of 21 (71%) were found to have further reduced MEDD at 2- to 3-year follow-up.

Mean average pain intensity in the current sample was low-moderate (M = 3.9, SD = 2.1, range = 0–8). Age inversely predicted change in pain intensity from baseline to 2- to 3-year follow-up and was included as a covariate in the final model.

The effect of time on change in pain intensity from baseline to 4 months to 2 to 3 years was not significant (*P* = 0.15); the reduction in average pain intensity from 4 months (mean = 4.22, SE = 0.37) to 2 to 3 years (mean = 3.74, SE = 0.60) was also not significant (*P* = 0.63). Similarly, the reduction in average pain intensity from baseline to 2- to 3-year follow-up was not significant (*P* = 0.16) (Fig. 1). Since baseline, 11 patients of the current sample of 21 (52%) experienced sustained pain reduction by the 2- to 3-year follow-up, and 12 patients of the current sample of 21 (57%) experienced further pain reduction from the 4-month follow-up to the 2- to 3-year follow-up. Five patients of the current sample of 21 (24%) people reported increased average pain intensity from baseline to the 2- to 3-year follow-up.

## 4. Discussion

To the best of our knowledge, our data provide the longest-term follow-up report for community-based voluntary prescription opioid tapering. Although limitations exist, we found sustained opioid dose reductions 2 to 3 years later and pain stability for a substantial fraction of patients who voluntarily enrolled in our original tapering study. These new results add to a growing body of knowledge on prescription opioid tapering methodology and outcomes. Recent studies have highlighted critical health risks associated with opioid reduction.^5,6,10^ There is an urgent need to better characterize patient risks both proximal and distal to opioid reduction to improve opioid stewardship, mitigate iatrogenic harms, and optimize patient outcomes. Indeed, opioid tapering methodology has been a point of controversy among patients^1,11^ and the medical community,^4^ and the recent HHS opioid tapering guidance calls for application of voluntary opioid tapering methods whenever possible.^7^ Our original voluntary opioid reduction received considerable attention, and a persistent and heretofore unanswered question has been about the durability of effects for pain and reduced MEDD. Our current findings provide key information that begins to answer that question.

Many points bear consideration, and results should be viewed within the context of several important limitations. First, we captured close to half of our initial study sample, and we highlight a selection bias associated with responders. Possibly, those who responded to our survey were experiencing better long-term outcomes, and thus, we are failing to capture the experience of patients who do poorer over time. Accordingly, we encourage a conservative interpretation of our data: A fraction of patients do quite well with opioid reduction long-term, and much remains to be learned.

Second, we underscore that these results do not generalize to nonconsensual opioid tapering as we studied voluntary tapering only. Third, 5 patients (24%) had poor long-term outcome in terms of increased pain. Future research should focus on meeting the needs of patients who report increased pain with opioid reduction, and we recognize that opioids may be an essential part of their care plan.

Fourth, because of changes in medical care, we were not able to verify current opioid use through medical records, and study data are limited by self-report. A fifth and final consideration is the brief patient assessment, which was a strategic effort to increase patient engagement and data collection. However, our ultrabrief survey precluded our ability to determine whether other pain management or nonpharmacological interventions were trialed by patients in the intervening timeframe.

Despite the aforementioned limitations, our brief report provides evidence for continued opioid reduction and enduring pain stability for some patients. Our low-cost, scalable, and patient-centered voluntary opioid tapering methods seem to have long-term efficacy for a substantial fraction of patients, a point that may offer hope and reassurance to many patients who may wish to reduce prescriptions but are ambivalent because of a lack of long-term outcomes data. Finally, we strongly underscore the need to better characterize and treat patients who do poorly with voluntary opioid tapering and for whom opioid reduction may be contraindicated and/or other pain management strategies are required.

## Disclosures

The authors have no conflicts of interest to declare.
